# MRI-based deep transfer learning models for predicting progesterone receptor expression in meningioma

**DOI:** 10.3389/fonc.2025.1517205

**Published:** 2025-03-31

**Authors:** Song Gao, Li Zhao, Nan Li, Xiaoming Zhou, Chongfeng Duan

**Affiliations:** ^1^ Department of Radiology, The Affiliated Hospital of Qingdao University, Qingdao, China; ^2^ Department of Information Management, The Affiliated Hospital of Qingdao University, Qingdao, China

**Keywords:** meningioma, progesterone receptor, magnetic resonance imaging, deep transfer learning, predict

## Abstract

**Objectives:**

The progesterone receptor (PR) is an important biomarker in meningiomas, influencing tumor growth, prognosis, and potential treatment options. The objective of this study was to predict PR expression in meningioma via deep transfer learning (DTL).

**Methods:**

A total of 307 patients were included in the study, including 173 positive patients and 134 negative patients. The clinical features were analyzed. The DTL features were extracted via the fine-tuned ResNet 50 model and selected by the intraclass correlation coefficient (ICC), spearman correlation coefficient and least absolute shrinkage and selection operator (LASSO). The predictive models were built via logistic regression (LR), support vector machine (SVM) and naive Bayes. The discriminative ability of the model was assessed by receiver operating characteristic (ROC) curve analysis and the area under the curve (AUC). The accuracy, sensitivity and specificity were also calculated. Decision curve analysis (DCA) curves were drawn to evaluate the clinical usefulness of the nomogram.

**Results:**

A total of 2048 DTL features were extracted, and 35 features were selected for model construction. In the test set, the AUCs of the LR, naive Bayes, and SVM models were 0.819 (95% CI: 0.7081-0.9300), 083(95% CI: 0.7216-0.9376), and 0.842 (95% CI: 0.7359-0.9488), respectively. There was no significant difference between any two models according to the Delong test. The SVM model exhibited a greater net benefit across the highest probability according to the DCA curve.

**Conclusions:**

The SVM model achieved better predictive performance and represents a useful tool for evaluating meningioma.

## Introduction

Meningioma is a common tumor with the highest incidence among all primary central nervous system tumors (1–3). Meningioma is categorized into three subtypes, namely, Grade 1, Grade 2, and Grade 3, based on the 2021 classification of the World Health Organization (WHO) (1). Different grades of tumors present varying biological behaviors and prognoses (4, 5) and the prediction of tumor grade is critical for clinical decision-making (4, 6, 7). However, other valuable evaluation indices, in addition to the WHO grade, have been insufficiently examined in meningioma studies. The progesterone receptor (PR) plays a significant role in the development and progression of meningioma. The PR is an important biomarker in meningioma, influencing tumor growth, prognosis, and potential treatment options.

The expression of PR has been found to affect the growth of meningioma (8–11). Moreover, the behavior of meningioma varies between sexes, with a higher incidence in females than in males, particularly during reproductive years. This suggests a potential link between sex hormones and tumor growth. Additionally, meningioma tends to grow rapidly in pregnant women or patients receiving hormone replacement therapy. Interestingly, grade 2 and 3 meningioma are more common in males. Several studies have also shown a correlation between PR expression, WHO grade, and recurrence (12–16). Specifically, negative PR expression is associated with a greater risk of recurrence and more aggressive tumors. PR positive meningiomas tend to be less aggressive and more responsive to treatment. Higher PR expression is associated with lower recurrence rates, especially in WHO Grade 1 meningiomas. The PR can also influence the potential treatment options. Progesterone antagonists could be potential treatment options and PR status may help guide post-surgical management and the need for additional therapy. Therefore, PR expression is a valuable biomarker for evaluating meningioma and warrants further investigation.

Although convolutional neural networks (CNNs) are widely used in deep learning and rely on real-world images, constructing a CNN medical model can be challenging due to the limited sample sizes in medical research. As a result, deep transfer learning (DTL) has become a popular approach in various studies. DTL involves training a pretrained model on a vast dataset, such as ImageNet, to detect a broad range of image features. These features can then provide a foundation for other image-related tasks. By fine-tuning the pretrained model on a smaller dataset specific to the task and teaching it to recognize new features, its performance on a new task can be improved (17).

Several previous studies have employed DTL to predict the grade of meningioma (18, 19). However, to the best of our knowledge, no study has yet utilized DTL to predict PR expression in meningiomas. In this study, with the aim of providing a more comprehensive evaluation of the disease and offering valuable support for clinical decision-making, we employed DTL to predict PR expression in meningioma.

## Materials and methods

### Patients

This study was performed in accordance with the Declaration of Helsinki. Approval for this retrospective study from the institutional review board of the Affiliated Hospital of Qingdao University was acquired, and the requirement for informed consent was waived by the institutional review board of the Affiliated Hospital of Qingdao University. We searched for patients who were diagnosed with meningioma from January 1, 2012, to January 1, 2022, in the pathology database. The patients were selected according to the following inclusion criteria: (1) did not receive any treatment before surgery, (2) magnetic resonance imaging (MRI) examination was applied one week before surgery, and (3) tumor grade and PR expression were given during the pathological diagnosis. The exclusion criteria were as follows: (1) the image quality was affected by severe artifacts and was not suitable for analysis, and (2) the tumor grade or PR expression was ambiguous. The enrolled patients were divided into positive and negative PR expression groups. There were 307 patients enrolled in the study, including 173 positive patients and 134 negative patients. The clinical data, including sex and age, were recorded. The training set and the test set were randomly divided at a ratio of 8:2.

### MRI examination and MRI feature analysis

Signa 1.5T and 3.0T MRI from GE and prisma and skyra 3.0T MRI from Siemens were used with the same scan parameters as follows: TR=1800 ms; TE=10 ms; slice thickness=5 mm; and FOV=25 cm. Each patient received 0.1 ml/kg Gd-DTPA before enhanced T1WI examination. The enhanced T1 WI images were used for further analysis.

Two neuroradiologists with 10 years and 20 years of experience analyzed the images. Both of the neuroradiologists were blinded to the group of tumors and interpreted the following MRI features by consensus: size, shape, heterogeneous enhancement, necrosis or cystic degeneration, indistinct margins, peritumoral edema, and surrounding invasion.

### Image preprocessing and tumor segmentation

**Figure 1:**
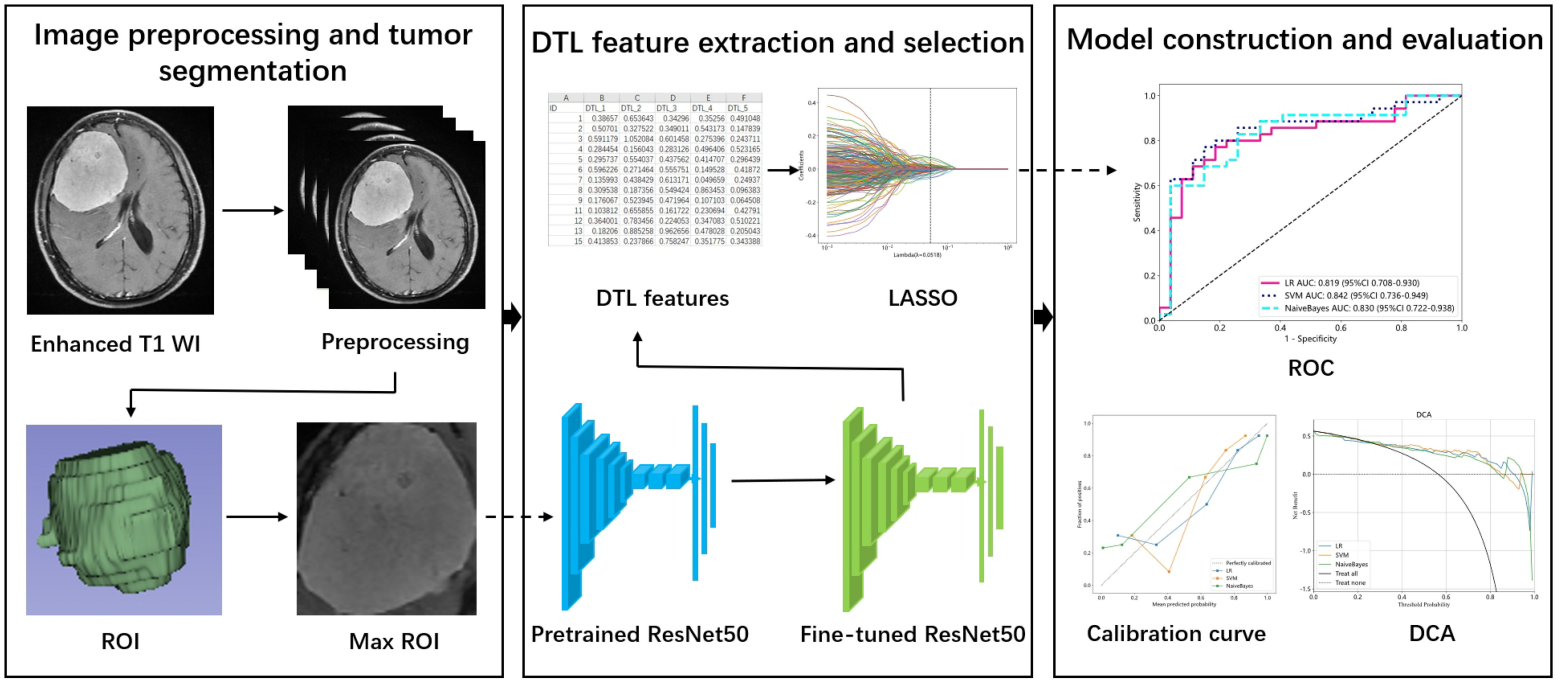
The workflow of the study. ROI, Region of interest; DTL, Deep transfer learning; LASSO, Least absolute shrinkage and selection operator; ROC, Receiver operating characteristic; DCA, Decision curve analysis.

The workflow of the study is shown in **Figure 1**. Image preprocessing and tumor segmentation were accomplished via 3D slicer software (version 4.11, https://www.slicer.org/). The image preprocessing procedure of enhanced T1 WI images included three procedures: N4ITK MRI bias correction, bin width, and image resampling. To eliminate unwanted low-frequency intensity nonuniformity, the N4ITK MRI bias correction method was employed (20). A uniform bin width of 25 was applied to normalize the image intensities across various MRI machines. The original resolution of the images was 0.5 × 0.5 × 5mm³. The image was resampled to achieve a voxel size of 1 × 1 × 1 mm³ by the algorithm of nearest neighbor interpolation, ensuring the preservation of scales and directions (21).

The processed enhanced T1 WI images were used to outline the region of interest (ROI). The ROI was carefully drawn by a neuroradiologist with 10 years of experience. The ROI contained all the slices of the tumor.

### DTL feature extraction and selection

We first cropped the max ROI from the 3D ROI of enhanced T1 WI images as the input to the model. It meant that one slice of the 3D volume with maximal diameter was utilized for feature extraction. Considering that the different MRI features of tumors (such as necrosis or cystic degeneration, calcification) determined the DTL features, all parts of the tumor were remained in the ROI. The max ROIs were normalized to the pixel of 224×224 by the algorithm of nearest neighbor interpolation. ResNet50 was selected as the pretrained model and all its layers were utilized. The images of the training and test sets were fed into the pretrained model for fine-tuning. The batch size and number of epochs were 32 and 30, respectively, in the fine-tuned model. All the images were input into the fine-tuned model to extract DTL features.

The stable features were selected by calculating the intraclass correlation coefficient (ICC). Twenty meningiomas were randomly selected from the dataset for ICC calculation. Two neuroradiologists independently drew the ROIs of the 20 meningiomas. The level of stable feature was set at higher than 0.80. The stable features were analyzed via the following methods.

Z score normalization was employed for the DTL features. The spearman correlation coefficient was used to evaluate the correlation between two features. Only one feature was retained if the correlation coefficient of two features was >0.9. Finally, we utilized least absolute shrinkage and selection operator (LASSO) to select features for model construction.

### Model construction and evaluation

The machine learning models included logistic regression (LR), support vector machine (SVM) and naive Bayes. The performances of different models were compared. The discriminative ability of the model was assessed by the receiver operating characteristic (ROC) curve and area under the receiver operating characteristic curve (AUC). The accuracy, sensitivity and specificity were also calculated. The calibration curve was used to assess the calibration of the model. Decision curve analysis (DCA) curves were drawn to evaluate the clinical usefulness of the nomogram.

### Statistical analysis

The categorical variables were compared via the chi-square test, whereas the quantitative variables were compared via the t test to evaluate differences between two groups. A DeLong test of AUCs was performed to assess the models.

p < 0.05 was considered statistically significant. The data processing was based on the one-key AI platform (http://www.medai.icu). The codes can be found at https://gitee.com/wangqingbaidu/OnekeyCompo?_from=gitee_search.

## Results

**Table 1** presents the clinical characteristics of the two groups in the training and test sets. The numbers of positive and negative patients in the training and test sets were 139 and 106 and 34 and 28, respectively. There was no significant difference in the clinical characteristics of the patients in the training set. There was a significant difference in sex in the test set.

**Table 1:** The clinical features of the two groups in the training and test sets.

	Training set			Test set		
	NEG	PEG	P	NEG	PEG	P
Age (year)	55.63±12.01	56.06±11.00	0.77	52.54±13.99	55.82±10.75	0.299
Size (mm)	50.44±17.07	48.41±19.61	0.396	49.28±20.58	48.27±22.51	0.856
Sex (female: male)	74:32	88:51	0.353	16:12	29:5	0.029
Shape (regular: unregular)	62:44	91:48	0.325	18:10	25:9	0.611
Heterogenous enhancement (absent: present)	72:34	88:51	0.538	17:11	24:10	0.584
Necrosis or cystic degeneration (absent: present)	86:20	107:32	0.529	22:6	28:6	0.958
Indistinct margin (absent: present)	87:19	114:25	1	22:6	28:6	0.958
Peritumoral edema (absent: present)	46:60	76:63	0.105	14:14	20:14	0.661
Surrounding invasion (absent: present)	84:22	113:26	0.812	23:5	29:5	1

NEG, negative expression group; PEG, positive expression group.

**Figure 2:**
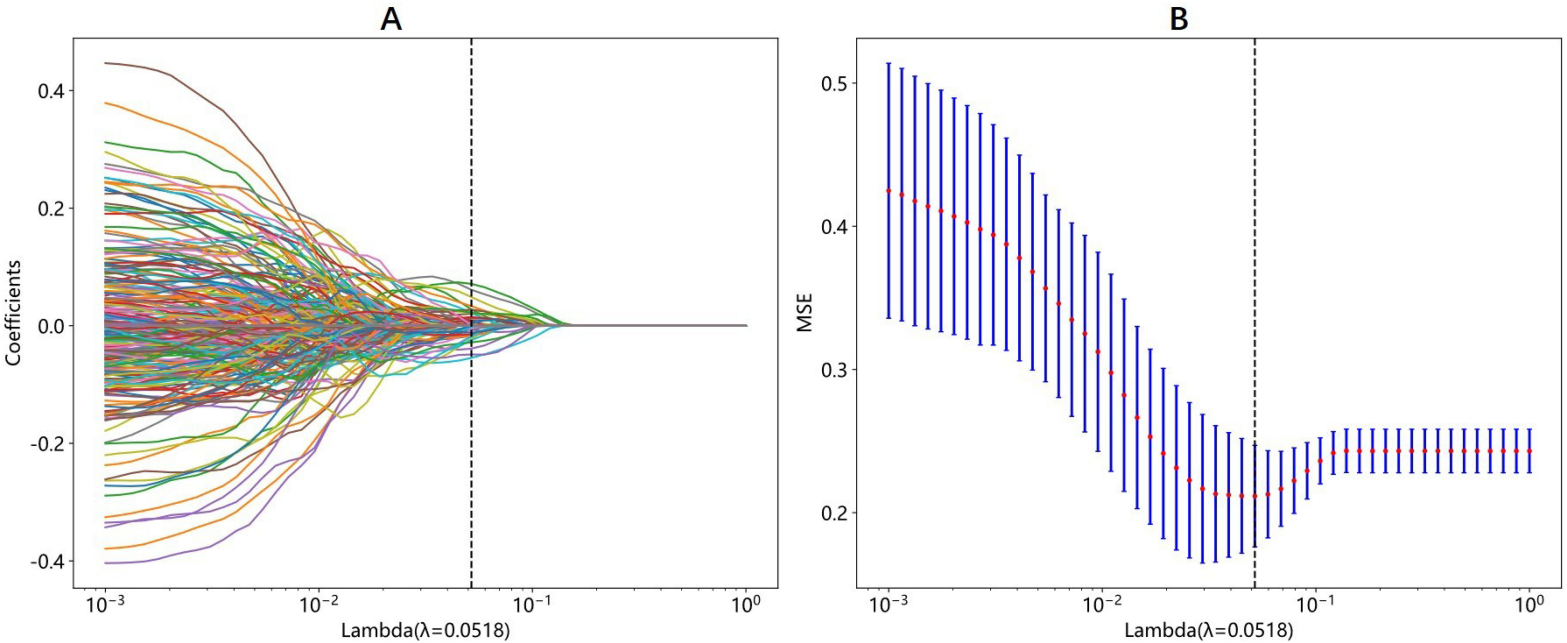
The process of LASSO regression in DTL features selection. **(A)** Different color line represents corresponding coefficient of each feature. **(B)** Tuning parameter(λ) selection in LASSO. The λ value of 0.0518 is selected.

**Figure 3:**
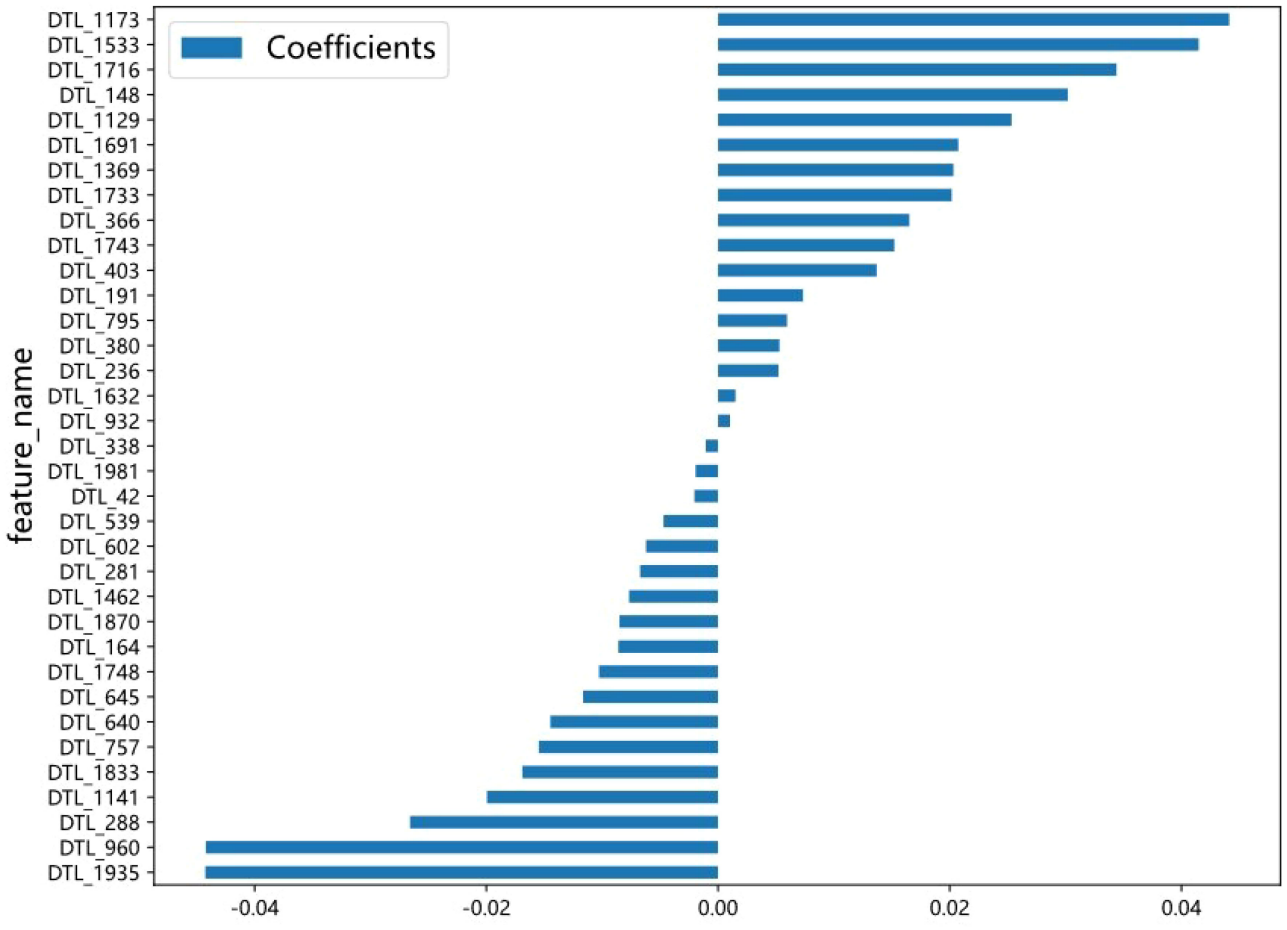
The selected DTL features and the correlation coefficient. The X axis represents the coefficient, and the Y axis represents the feature name. The length of the blue bar chart represents the coefficient value of the feature.

**Figure 4:**
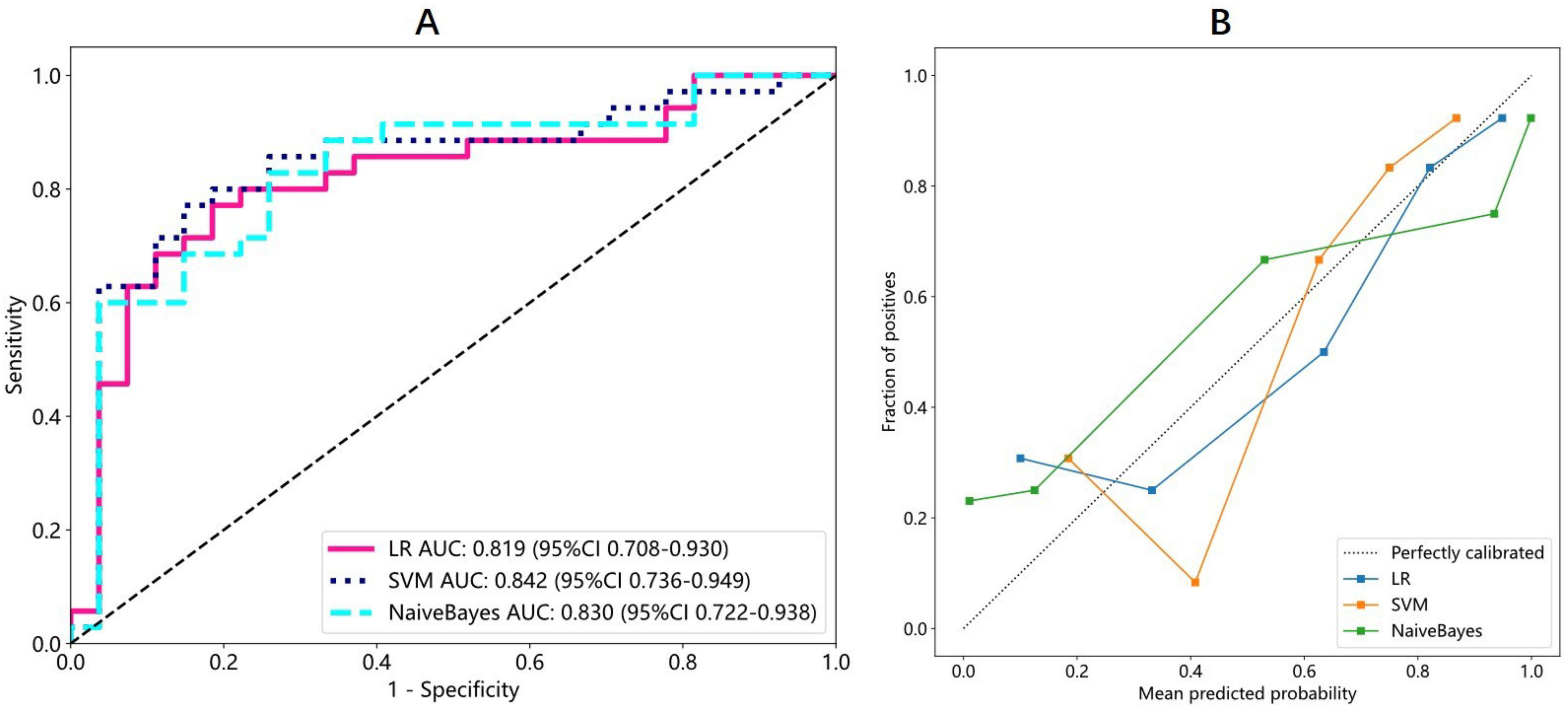
The ROC **(A)** and calibration curve **(B)** of LR model, SVM model and Naive Bayes model. **(A)** The AUCs of the LR, naive Bayes, and SVM models were 0.819 (95% CI: 0.7081-0.9300), 0.83(95% CI: 0.7216-0.9376), and 0.842 (95% CI: 0.7359-0.9488) in the test set. SVM model achieved the highest AUC. **(B)** In the calibration curve, the 45° straight line represents the perfect match between the actual and predicted probabilities. The closer the curve approaches perfectly calibrated line, the better the calibration of the model is. Three models both have a good calibration. LR, Logistic regression; SVM, Support vector machine.

**Figure 5:**
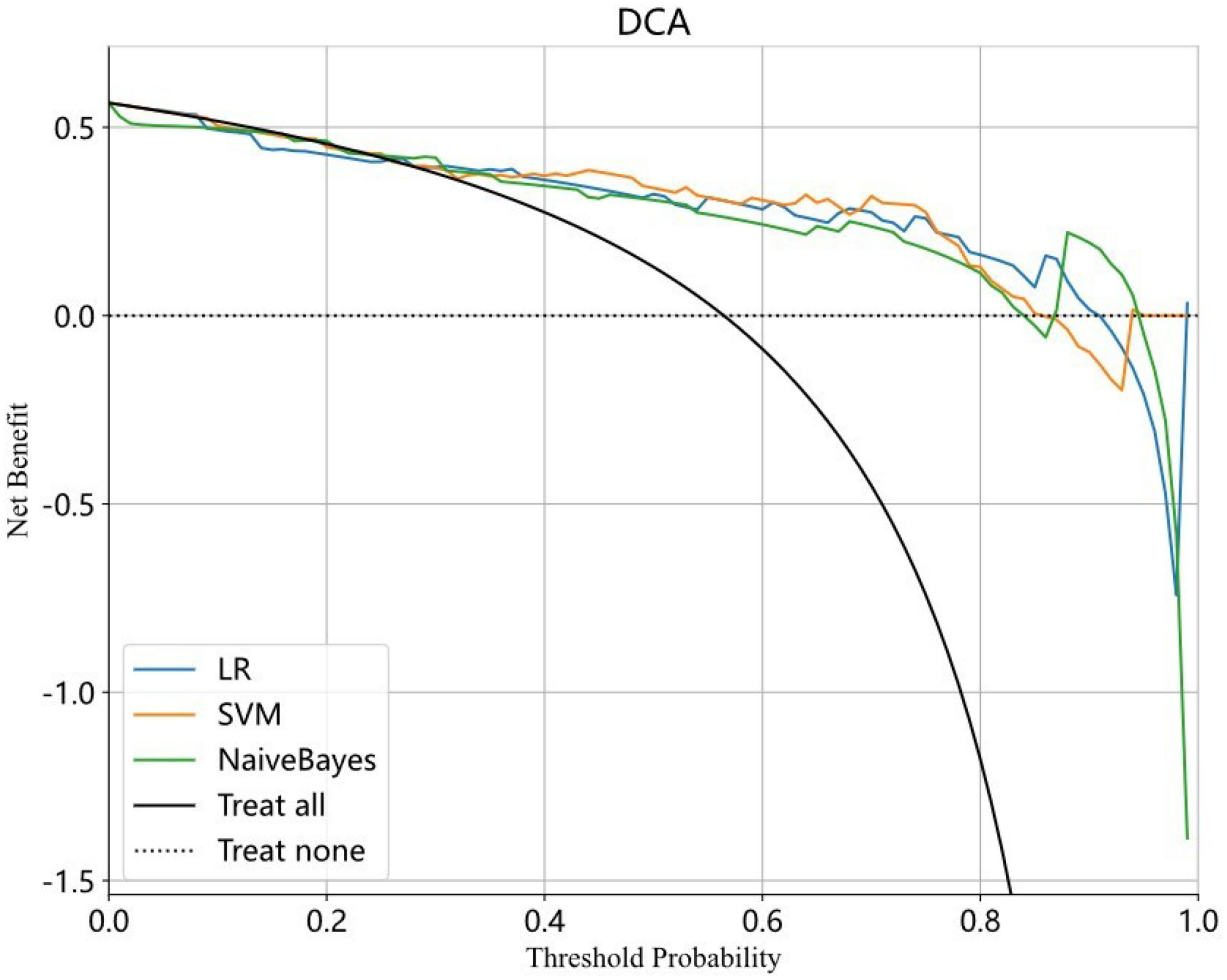
The DCA curve of LR model, SVM model and Naive Bayes model. The X axis represents the threshold probability and the Y axis represents the net benefit. The blue, yellow and green curve respectively represent LR, SVM and Naive Bayes model. The SVM model achieves greater net benefit than the LR and naive Bayes models across the most range probability. The SVM model has a better clinical utility. LR, Logistic regression; SVM, Support vector machine.

A total of 2048 DTL features were extracted in each ROI from the fine-tuned model. Thirty-five features were selected after ICC calculation, spearman analysis and LASSO regression. The dimensionality reduction process of LASSO is shown in **Figures 2**, **3** shows the selected features and their correlation coefficients. The λ value of LASSO was 0.0518. **Table 2**; **Figure 4A** show the results of the different models. The AUCs of the LR, naive Bayes, and SVM models were 0.899 (95% CI: 0.8618-0.9360), 0.861 (95% CI: 0.8150-0.9076), and 0.974 (95% CI: 0.9592-0.9896) in the training set and 0.819 (95% CI: 0.7081-0.9300), 0.83 (95% CI: 0.7216-0.9376), and 0.842 (95% CI: 0.7359-0.9488) in the test set, respectively. There was no significant difference between any two models in the Delong test (LR vs. SVM=0.2899, LR vs. naive Bayes=0.7388, SVM vs. naive Bayes=0.5917). **Figure 4B** shows the calibration curves of the model. According to the DCA curve (**Figure 5**), the SVM model had a greater net benefit across the highest probability than the LR and naive Bayes models did.

**Table 2:** The results of different models.

	AUC (95% CI)	Accuracy	Sensitivity	Specificity	PPV	NPV
Training set						
LR	0.899 (0.8618 - 0.9360)	0.816	0.804	0.832	0.86	0.767
Naive Bayes	0.861 (0.8150 - 0.9076)	0.796	0.819	0.766	0.819	0.766
SVM	0.974 (0.9592 - 0.9896)	0.922	0.964	0.869	0.905	0.949
Test set						
LR	0.819 (0.7081 - 0.9300)	0.79	0.771	0.815	0.844	0.733
Naive Bayes	0.83 (0.7216 - 0.9376)	0.79	0.829	0.741	0.806	0.769
SVM	0.842 (0.7359 - 0.9488)	0.806	0.771	0.852	0.871	0.742

LR, Logistic regression; SVM, Support vector machine; AUC, Area under the receiver operating characteristic curve; CI, Confidence interval; PPV, Positive predictive value; NPV, Negative predictive value.

## Discussion

The present study constructed three models to predict PR expression in meningioma, each of which exhibited good predictive performance with a relatively high AUC. Among the models, the SVM model demonstrated superior performance, as evidenced by a greater net benefit across the highest probability in the DCA curve. Specifically, the SVM model achieved an AUC of 0.842 (95% CI: 0.7359-0.9488), with an accuracy rate of 0.806, a sensitivity value of 0.771, and a specificity value of 0.852 in the test set.

The role of PR expression in meningioma growth and prognosis has been extensively studied. Numerous studies have shown that meningiomas with higher grades, greater cellular proliferation, and a greater risk of recurrence are more likely to have negative PR expression. Kuroi Y et al. (22) reported that negative PR expression was correlated with recurrence and shorter recurrence-free survival in meningioma patients. Furthermore, their study revealed a significantly greater proportion of positive PRs in skull base meningiomas than in nonskull base meningiomas. Maiuri F et al. (23) reported an inverse correlation between PR expression and WHO grade and Ki67-MIB1 expression (p < 0.0001). They also reported that low or moderately low PR expression was significantly correlated with recurrence (p = 0.0004). Despite the importance of PR expression in meningioma, only a few studies have attempted to predict PR expression preoperatively. Bozdağ M et al. (24) assessed the relationships between the apparent diffusion coefficient (ADC) and several histopathological parameters, but no significant correlation between the ADC and the PR score was observed.

The present study was the first to construct DTL models to predict PR expression. DTL is a powerful machine learning technique that leverages a pretrained model to address new tasks. By transferring the pretrained model’s knowledge to the new task, DTL enables the model to achieve higher performance with less data and computational resources. DTL has been widely used in various fields, such as computer vision, natural language processing, and speech recognition. In meningioma research, DTL has been primarily employed for grade prediction. In our study, we used DTL to predict PR expression and achieved good predictive performance. The SVM model had an AUC of 0.842 (0.7359-0.9488), with an accuracy rate of 0.806, a sensitivity value of 0.771 and a specificity value of 0.852 in the test set. The SVM model had a greater net benefit across the highest probability according to the DCA curve. DCA is a valuable method for evaluating the clinical utility of predictive models by assessing their net benefit across different threshold probabilities. It helps determine whether using a model to guide clinical decisions would improve outcomes compared to alternative strategies. The model with the highest net benefit across a range of threshold probabilities is considered the most clinically useful. In the present study, the SVM model achieved greater net benefit than the LR and naive Bayes models across the highest probability in DCA.

The present study had several limitations that need to be acknowledged. First, the retrospective nature of the study may have introduced selection bias and overestimated the accuracy of the diagnosis. Second, the DTL features were extracted solely from enhanced T1WI, which does not include other important MRI sequences, such as T1WI, T2WI, FLAIR imaging, and DWI. Thus, future studies should consider the use of multiple sequences. Third, the max slice of ROI was utilized for prediction instead of entire 3D ROI due to computational complexity and limited hardware resources. Considering that 3D ROI contained more tumor information, we will use 3D ROI to improve prediction accuracy in the future. Fourth, although the sample size was relatively large, further validation of the results in more meningiomas and other centers is necessary before clinical application. Moreover, although SVM model achieved a high AUC, there was no significant differences between the models in the Delong test. Multicenter and large sample studies are needed to improve the robustness of the findings. To address this, we plan to increase the number of cases in future studies.

## Conclusion

We developed three models for predicting PR expression in meningiomas and achieved better predictive performance. Among the three models, the SVM model performed the best, with an AUC of 0.842 (95% CI: 0.7359–0.9488), an accuracy rate of 0.806, a sensitivity value of 0.771, and a specificity value of 0.852. Our study provides valuable insights for assessing meningiomas and offers practical recommendations to aid in clinical decision-making.

## Data Availability

The raw data supporting the conclusions of this article will be made available by the authors, without undue reservation.
